# Terpene-Derived Low-Viscosity Deep Eutectic Solvent for Energy-Efficient CO_2_ Absorption

**DOI:** 10.1021/acsomega.5c06033

**Published:** 2025-09-30

**Authors:** Sinchan Hait, Upasana Mahanta

**Affiliations:** Department of Chemical Engineering, 166231 Birla Institute of Technology and Science, Pilani, K.K. Birla Goa Campus, Zuarinagar, Sancoale, Goa 403726, India

## Abstract

Deep eutectic solvent (DES) is considered an attractive solution for mass transfer operations due to its favorable vapor pressure and physicochemical properties. This study investigated the CO_2_ absorption capacity of a type V hydrophobic natural DES (HNDES) having camphor (CAM) and menthol (MEN) as HBAs and HBDs, respectively. The HNDES was prepared at a 4:7 molar ratio and obtained from the Conductor-like Screening Model for Real Solvents. The properties, namely, viscosity, vapor pressure, boiling point, and flash point, were measured. The solvent’s viscosity was found to be 23 mPa.s at 298.15 K. The CO_2_ solubility was investigated at a temperature range of 298.15–323.15 K. The operating pressure was varied from 0.2 to 0.6 MPa. The highest mole fraction of CO_2_ in the solvent was observed to be 0.044 at 303.15 K and 0.6 MPa. The enthalpy of absorption (−14.7 kJ.mol^–1^) indicates a decrease in solubility at elevated temperature. Moreover, the CO_2_ physisorption was also validated by FTIR, Raman, and NMR analysis.

## Graphical abstract

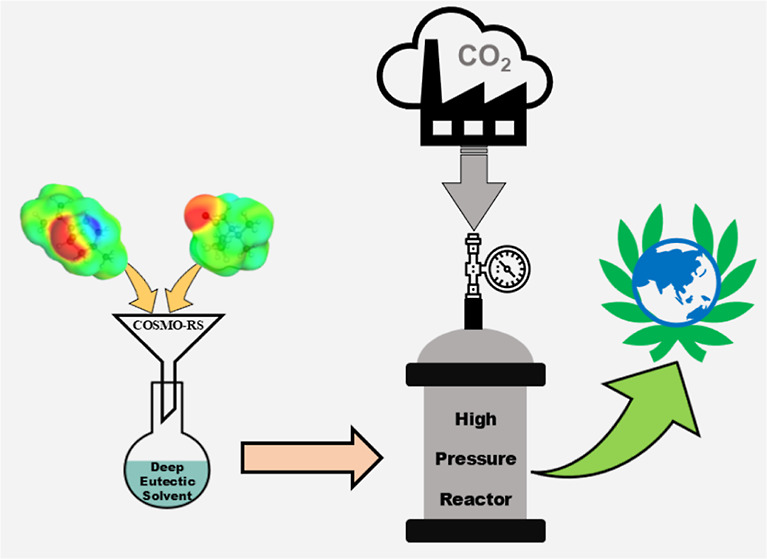

## 1 Introduction

CO_2_ emissions and their consequences have become a major concern worldwide with rapid technological advancements. CO_2_ is considered to be one of the primary causes for global warming and, thus, climate calamity.^1–3^ The well-known impacts of CO_2_ on health and environment motivate researchers, scientists, and engineers to develop sustainable technologies for carbon capture, utilization, and storage (CCUS). Technologies such as absorption, adsorption, membranes, cryogenic separation, gas hydrate crystallization, and direct air capture (DAC) have been developed to address this challenge.^4^ Solvent-based technologies are the most promising among the various technologies developed and deployed.^1,2^ This mass transfer operation is driven by the affinity of the solvent toward CO_2_ molecules, either by chemical or physical interactions. Subsequently, solvent regeneration can be achieved by breaking the bonds using high temperature or depressurization.^2^ Traditionally, amines or aqueous amines have been the common choice for the same. Amines can absorb the CO_2_ molecules efficiently due to the formation of carbamate and/or carbonate. However, solvent degradation remains a significant challenge, as amine degradation produces potentially toxic compounds for the environment.^2,5^ Additionally, the process is also energy-intensive.

Ionic liquids (ILs) were explored to solve the challenges associated with amines. ILs are nonflammable and have low vapor pressure, which makes them sustainable for high-temperature applications. CO_2_ solubility in ILs is mainly governed by the free volume and the interactions between CO_2_ and ions during absorption.^6^ The high cost and poor biodegradation limit ILs’ industrial applications despite their promising CO_2_ uptake capacity. Additionally, the IL synthesis process is energy-intensive and product purification is also a crucial step. In this regard, deep eutectic solvents (DESs) can be considered as a sustainable alternative to amines and ILs. DESs are a mixture of hydrogen bond donors (HBDs) and hydrogen bond acceptors (HBAs) and introduced by Abbott *et al.* ^7^ They remain as a liquid at room temperature, and their melting temperature (*T*_m_) is lower than that of HBDs and HBAs.^7^ DESs also inherit physicochemical properties similar to those of the ILs. The HBD and HBA can be chosen according to the desired properties of the solvent while keeping an optimal raw material cost. Since the discovery of DESs, research on fundamentals and applications has grown day by day. A large portion of the literature reported on DESs lacks the eutectic composition and phase diagram. Besides that, a certain composition remains solid under the working temperature. Therefore, Chen and Yu have coined a new terminology for a better understanding of DESs as low-melting mixture solvents (LoMMSs) and proposed six new classifications,^8^ namely (1) class I: ionic–ionic, (2) class II: molecular–molecular, (3) class III: ionic-molecular, (4) class IV: metallic–metallic, (5) class V: ionic-metallic, and (6) class VI: molecular-metallic. The DES reported in the present work belongs to class II. A few notable applications of the DESs are electrodeposition, electropolishing, battery technology, supercapacitors, metal extraction, heat transfer, biological transformation, biomass processing, gas adsorption, and pharmaceuticals.^9–12^

Absorption of CO_2_ by a DES, namely, Reline (choline chloride/urea—1:2), was originally reported by Li *et al*.^13^ DESs are made from natural sources, such as fatty acids, amino acids, sugars, terpenes, etc., and interest researchers.^1–3,14–18^ These DESs are termed natural deep eutectic solvents (NADES). Moreover, the current focus is on hydrophobic DESs to minimize the effect of water on CO_2_ absorption. Apart from that, various other parameters also hinder the CO_2_ solubility, such as solvent acidity, density, and viscosity. In this regard, Zubeir *et al.* reported a hydrophobic DES by combining tetrabutylammonium chloride (N_4444_–Cl), tetraoctylammonium bromide (N_8888_–Br), methyltrioctylammonium chloride (N_8881_–Cl), tetraoctylammonium chloride (N_8888_-Cl)­and methyltrioctylammonium bromide (N_8881_–Cl) as HBA and decanoic acid (DecA) as HBD.^15^ Among the various molar ratios tested, DecA:N_8888_–Cl (1.5:1) resulted in the highest CO_2_ solubility (*x*_CO_2__ = 0.3) at 2 MPa and 298.15 K. Another study by Al-Bodour *et al.* explored thymol (THY), menthol (MEN), carvone (CAR), and cineole (CIN)-based natural hydrophobic DESs.^3^ The authors discussed the influence of HBAs and HBDs on CO_2_ capture. Siani *et al.* investigated zwitterionic NADESs with oxalic acid, phenylacetic acid, and glycolic acid as HBD and *N,N,N*-trimethyl glycine (TMG) as HBA.^2^ They noted the highest solubility for TMG:phenylacetic acid (1:2), i.e., 45.5 mg of CO_2_/g of DES under 4 MPa and 313.15 K. The authors also discussed the effects of acid dissociation constant (*pK*_a_) and free volume of the DESs on CO_2_ capture capacity. An investigation exploring polyamine-based hydrophobic DES in combination with a terpene component, ([TEPA]­Cl:THY at 1:3), reported a CO_2_ solubility of 1.355 mol CO_2_/mol DES at 313.15 K and 101.3 kPa.^18^ However, a significant gap exists in the literature for establishing a hydrophobic natural DES (HNDES) for CO_2_ capture. In addition, only a few studies explore acidity’s influence on CO_2_ solubility.

COnductor-like Screening MOdel for Real Solvents (COSMO-RS) can also be used to predict different properties of a solvent and phase equilibria data. It is a statistical thermodynamic model that uses a σ-profile for various property calculations. The present study combines computational and experimental approaches to study CO_2_ solubility in a terpene-derived HNDES, composed of menthol (MEN) and camphor (CAM). Padilla *et al.* reported that MEN and CAM could form a DES at a molar ratio of 2:1.^19^ The authors investigated thermophysical properties and lidocaine solubility in various terpene-based DESs, including CAM:MEN. In this work, first, COSMO-RS is used to identify the eutectic composition of the CAM and MEN. This thermodynamic model was widely adopted by researchers to obtain the eutectic composition of the DESs.^20–22^ COSMO-RS has been proven as a promising tool for predicting phase equilibria data.^16,23,24^ In addition, the vapor pressure, boiling point, and flash point of the HNDES and environmental parameters for HBA and HBD are also computed using COSMO-RS. Further, the CO_2_ solubility in HNDES is experimentally investigated, and the Henry’s law constant, enthalpy, and entropy of absorption are calculated.

## 2 Results and Discussion

### 2.1 σ-Profile and σ-Potential Analysis

COSMO-RS determines the probability distribution of surface screening charge density (SCD) on the molecular surface segment and that can be represented as a σ-profile (Figure 1a). The σ-profiles are divided as HBA zone (red, σ > +0.01 eÅ^–2^), HBD zone (blue, σ < −0.01 eÅ^–2^), and nonpolar zone (green, −0.01 eÅ^–2^ < σ < +0.01 eÅ^–2^), which also represents the sigma surface available for van der Waals interactions. It is observed that MEN can act as both HBA (+0.01 < σ < +0.02 eÅ^–2^) and HBD (σ < −0.01 eÅ^–2^), whereas CAM primarily possesses hydrogen bond accepting tendency (+0.01 < σ < +0.02 eÅ^–2^). Besides that, the peaks in the nonpolar region inform about the alkyl groups. Due to its structural symmetry, the σ-profile of CO_2_ falls into the nonpolar region. The σ-profile of the HNDES shows a wide peak in the central zone, indicating that primary intermolecular interaction is due to the van der Waals force. The HNDES shows its electrophilic nature with a stronger peak at σ > +0.01 eÅ^–2^.

**Figure 1:**
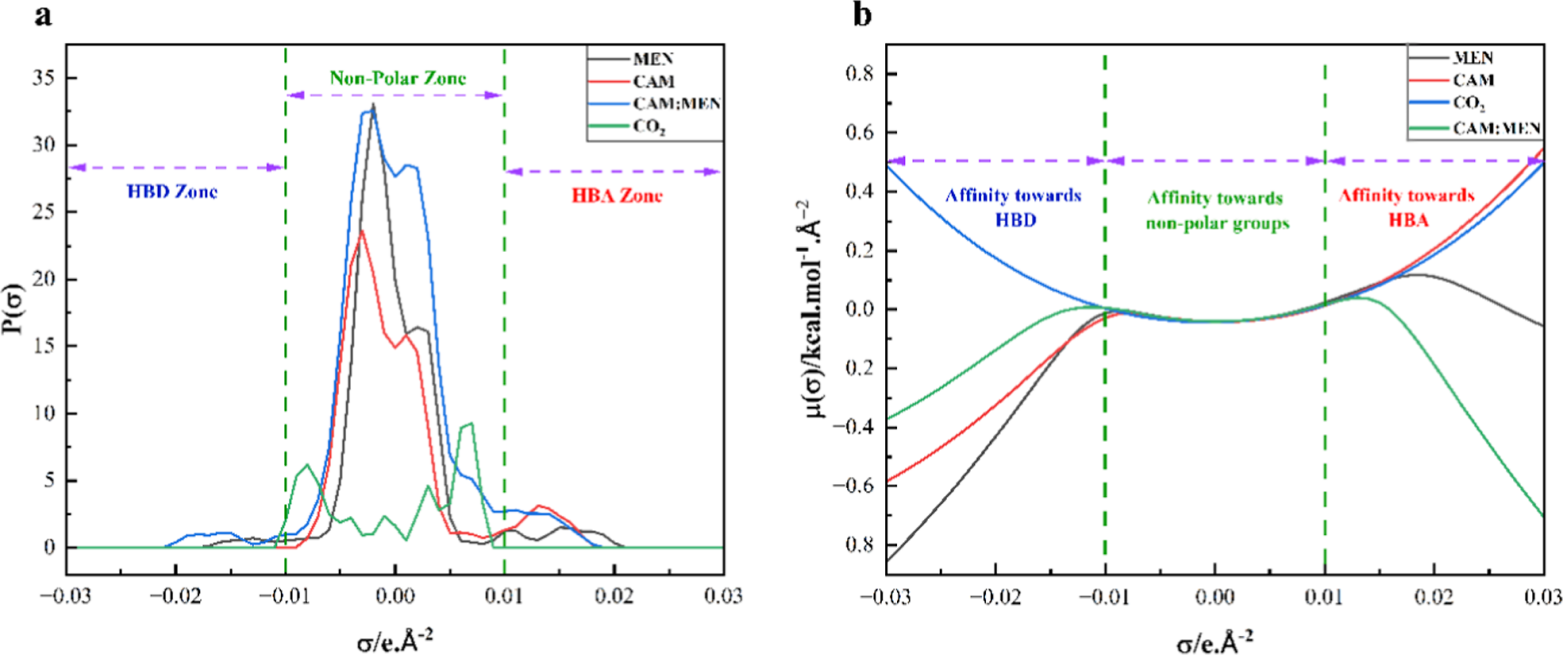
Sigma surface charge distribution calculation from COSMO-RS: (a) σ-profile and (b) σ-potential.

The formation of the DES requires hydrogen bonding between the two groups. The negative potential of the MEN and CAM is responsible for the H-bond formation (Figure 1b). The σ-potential of MEN confirms its affinity toward HBDs and slightly toward the HBAs. In addition, the HNDES σ-potential indicates its affinity toward both HBAs and HBDs. Also, from the surface charge distribution (Figure S1), it is prominent that the MEN can act as both HBD and HBA (blue and red regions). The CAM showed a greater affinity toward the HBD (Figure 1b). Similar observations have already been made from the σ-profile (Figure 1a). It can be concluded that a more negative potential of MEN results in a stronger interaction with CO_2_.

### 2.2 Eutectic Point Identification from COSMO-RS and DES Preparation

The phase equilibrium data of HBA and HBD can be viewed in Figure 2a. The mixture forms a eutectic solvent at a molar ratio of 4:7. Literature reported the same system with a molar ratio of 2:3, which is comparable to this work.^22^ The resulting HNDES possesses a significantly reduced melting point (291.78 K) compared to its counterparts (Table 1) due to hydrogen bond formation. Notably, the synthesis procedure does not require a supply of thermal energy, and the HNDES is formed in 2 h. This can be considered a significant achievement toward sustainability. The prepared solvent remains as a liquid at room temperature, as seen in Figure 2b.

**Figure 2:**
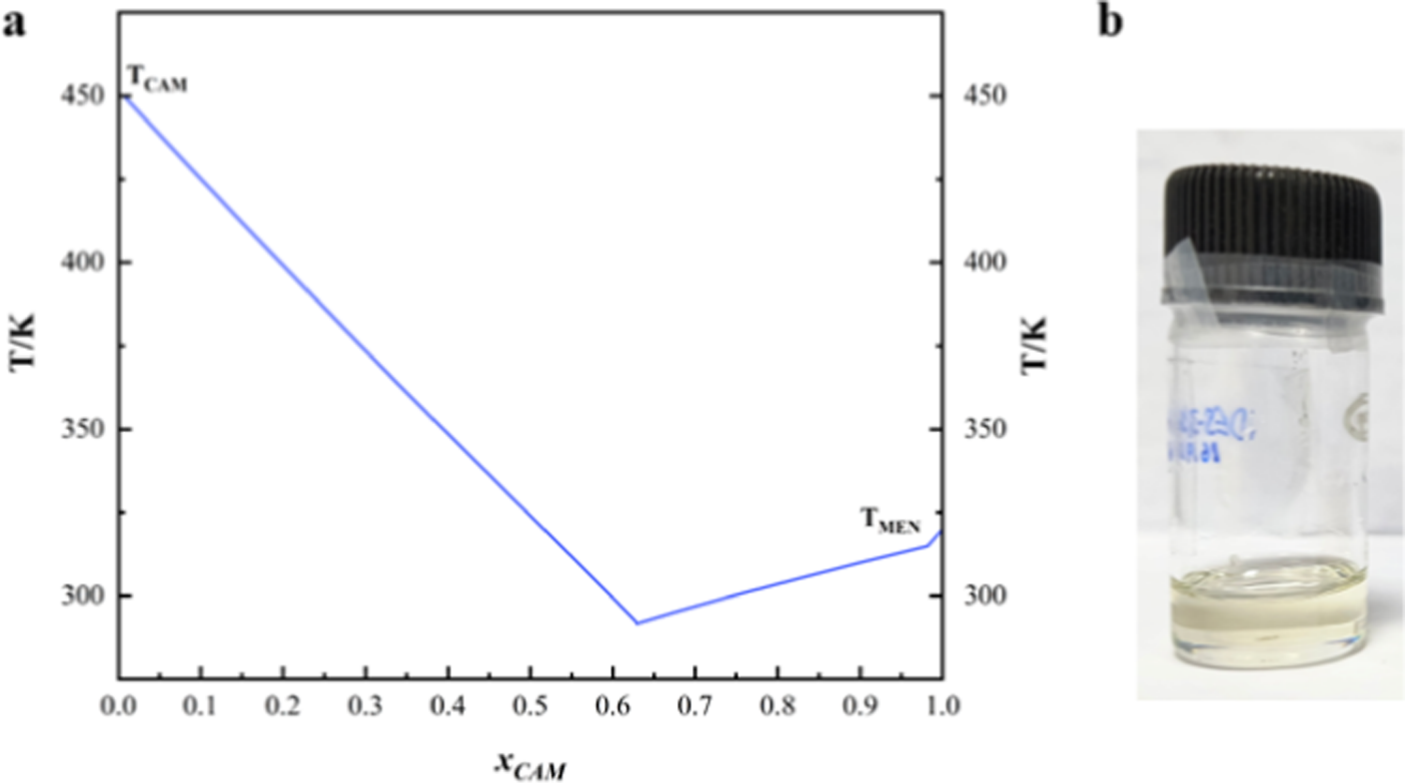
Prepared HNDES from COSMO-RS. (a) SLE plot from COSMO-RS computation. (b) Experimentally prepared HNDES by the mole fraction obtained from COSMO-RS.

**Table 1:** Thermodynamic Parameters and Model Used for the COSMO-RS Calculations

compound	*ΔH_fus_*/kJ.mol^–1^	*T*_m_/K	model
MEN^25^	14.1	316.05	DFT/B3-LYP/gridm3/DFT-D2 with BP_TZVP_24/COSMO-RS
CAM^26^	6.3	451.8	
CO_2_			

### 2.3 Physiochemical Analysis

#### 2.3.1 Viscosity and Other Physical Properties

High-viscosity liquids give rise to economic challenges regarding high pumping costs.^27,28^ In addition, the viscosity of the solvent is one of the determining parameters for the CO_2_ absorption. In this regard, low-viscosity solvents are preferred for gas absorption to accelerate the mass transfer rate.^27–29^ The viscosity of the HNDES was measured at a 293.15–317.15 K range (Figure 3). It is observed that the viscosity of HNDES decreases linearly with an increase in temperature. CAM:MEN is significantly low viscous compared to ILs and choline chloride (ChCl)-based DESs.^27,30,31^ ChCl:Urea (Reline) was reported to have a viscosity of 1600 mPa·s at 295.15 K, which is nearly 70 times higher than that of CAM:MEN (23 mPa·s at 298.15 K).

**Figure 3:**
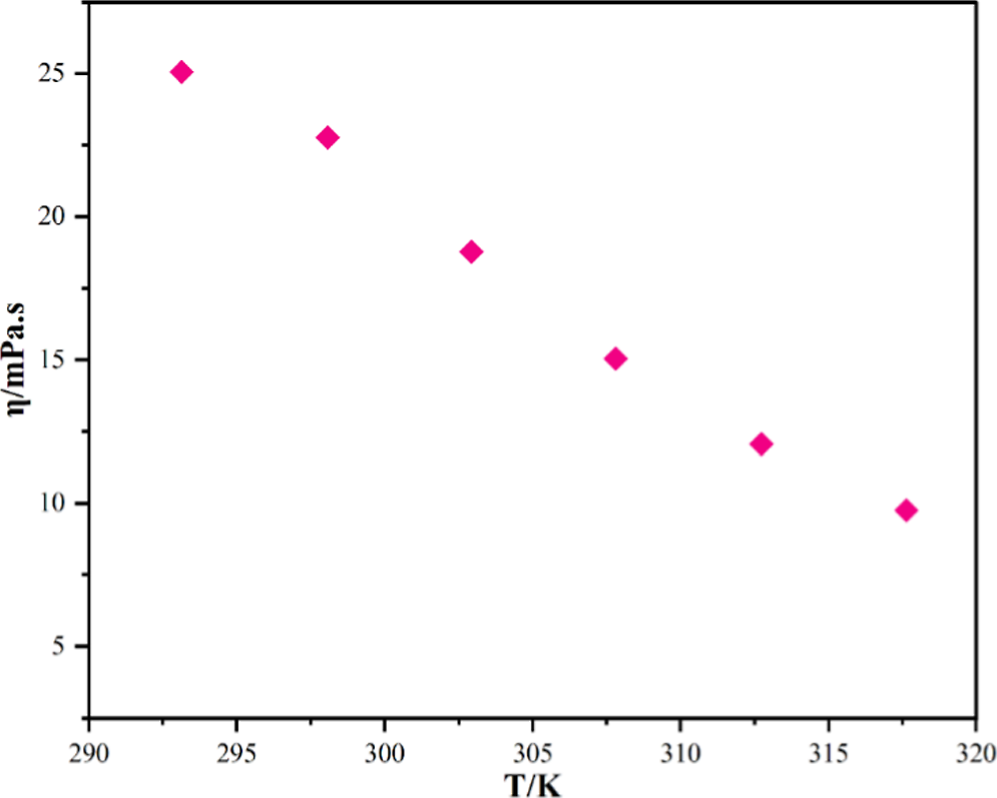
Viscosity of CAM:MEN at a temperature range of 293.15 to 317.15 K.

The knowledge of the solvent’s vapor pressure, boiling point, and flash point is crucial regarding industrial safety. The present work focused on predicting the aforementioned properties of HNDES using the COSMO-RS. Figure 4 depicts the predicted vapor pressure at a temperature range of 298.25 to 323.15 K. As expected, the vapor pressure exponentially rises with temperature, and at 298.15 K, the vapor pressure is 0.018 KPa. The computed values are comparable to those of other DESs, namely, Reline, Ethaline, and Glyceline.^32^ In addition, the boiling point and flash point of the solvent are found to be 496.47 and 351.27 K, respectively.

**Figure 4:**
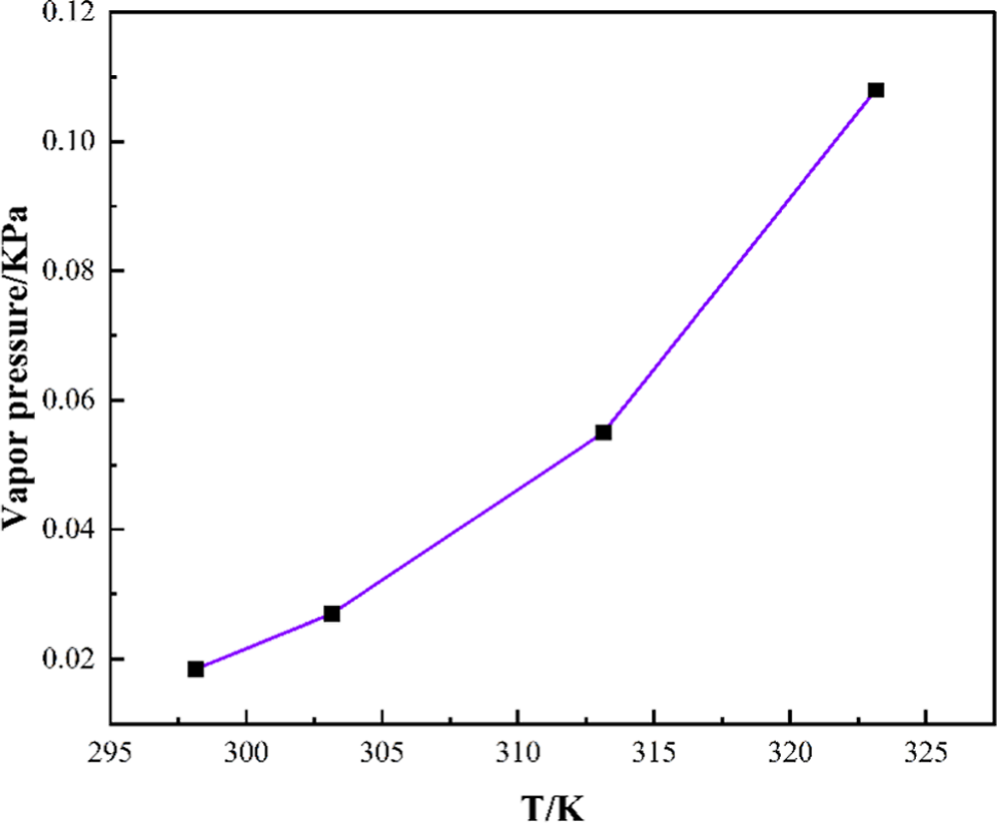
COSMO-RS predicted vapor pressure under 298.15 to 323.15 K.

#### 2.3.2 Thermal Degradation Studies

The system shows excellent thermal stability up to 323.15 K (Figure 5a) and thereafter starts losing mass with an increase in temperature. This marks 323.15 K as the maximum operating temperature of the DES at an industrial scale. This temperature is also termed the onset temperature (*T*_onset_), which signifies the beginning of mass loss. The studied DES loses its entire mass at around 399.15 K (*T*_offset_). In addition, the derivative thermogram (DTG) shows that maximum mass loss occurs between 375.15 and 395.15 K (Figure 5b). The DES possesses improved or equal thermal stability compared to previously reported MEN and THY-based DESs, except for CAR:THY and CIN:THY (Table 2).

**Figure 5:**
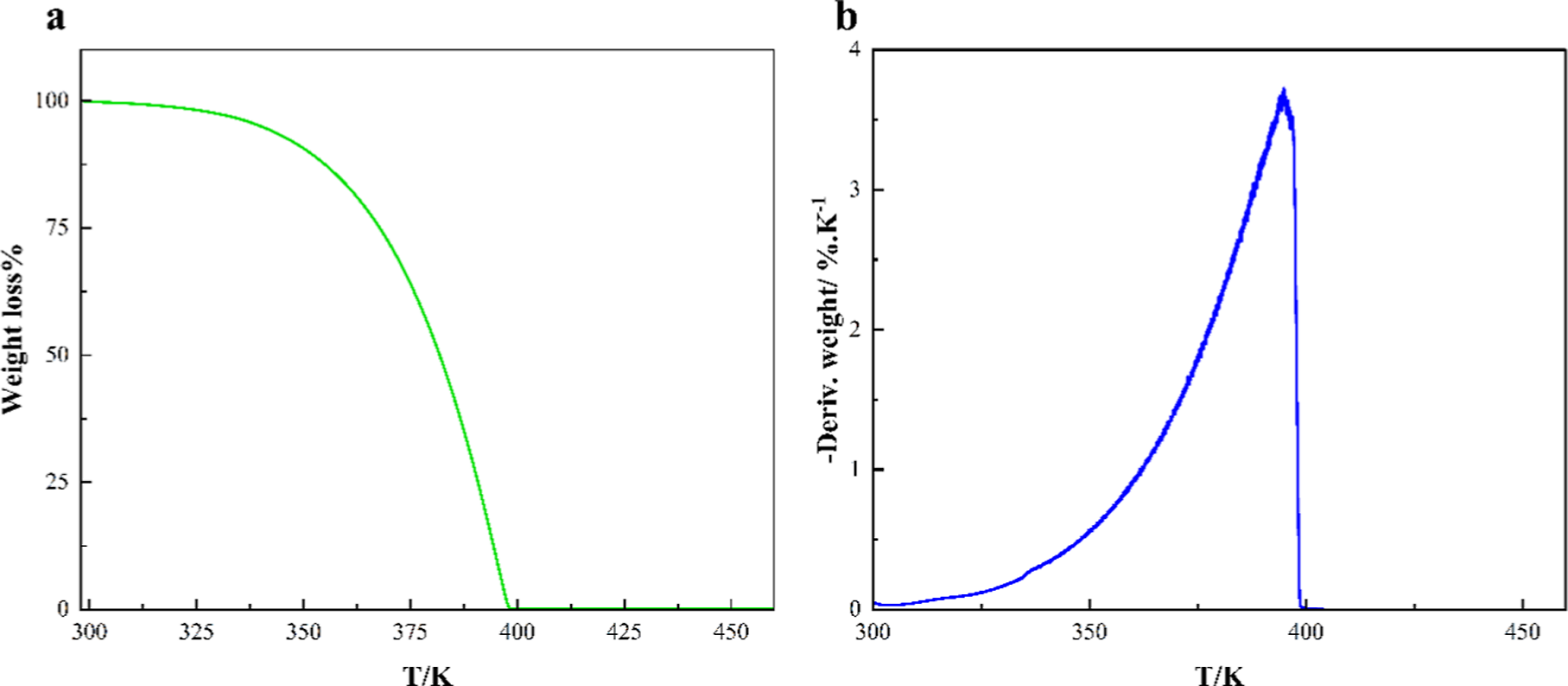
Thermal profiles of the DES: (a) % mass loss vs temperature and (b) derivative of % mass loss vs temperature.

**Table 2:** TGA Data Comparison

DES	*T*_onset_/K	*T*_offset_/K
CAM:MEN^[this study]^	323.15	399.15
CAR:MEN^3^	323.15	358.15
CIN:MEN^3^	313.15	365.15
CIN:THY^3^	358.15	384.15
CAR:THY^3^	343.15	403.15

#### 2.3.3 Acidity

The acidity of the solvent greatly influences the CO_2_ solubility. CO_2_–solvent interactions are stronger in the case of a basic solvent. The acidity of the present HNDES can be found in Figure 6 for a temperature range of 298.15 to 323.15 K. It is observed that with an increase in temperature, pH reduces, indicating an increase in the acidity of the solvent. Similar findings are also observed elsewhere.^33,34^ Hence, it can be concluded that the CO_2_ solubility is reduced at elevated temperatures. It is observed that the percent change in the CO_2_ solubility is much higher compared to that in acidity. This can be further understood from the negative change in enthalpy during absorption, as discussed in Section 2.5. Mirza *et al.*, studied different HBDs (urea, malic acid, and ethylene glycol), with one HBA.^1^ This study showed that the choice of HBD plays a crucial role in determining the acidity of a DES.

**Figure 6:**
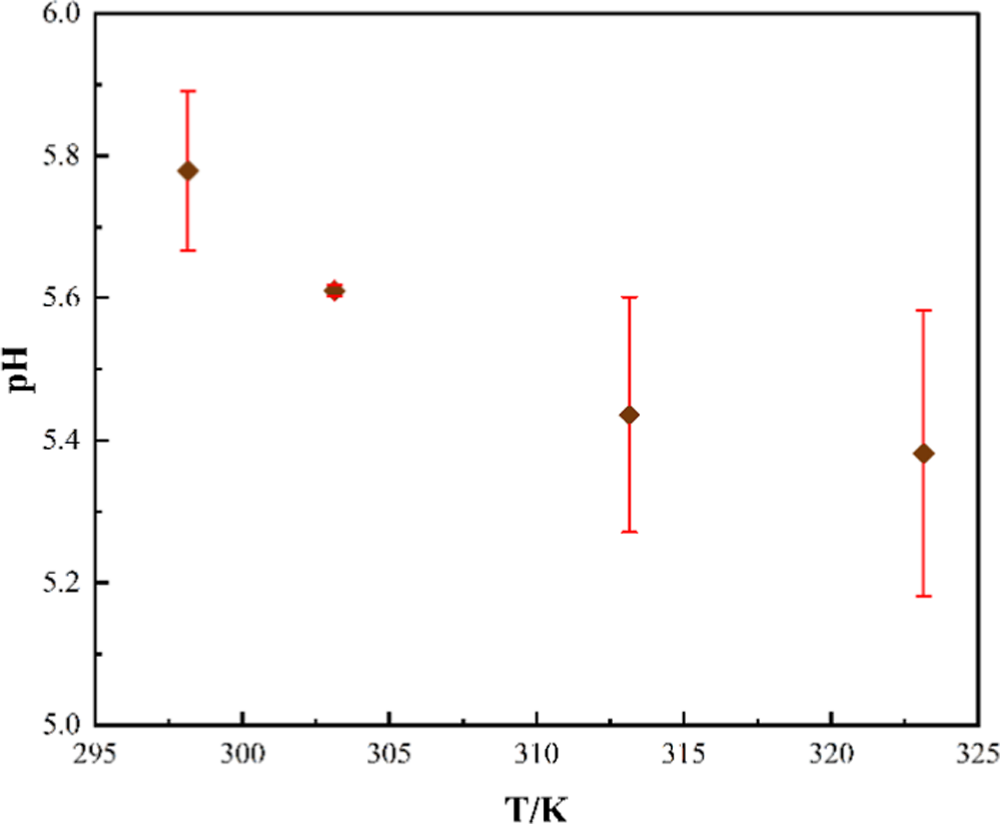
Effect of temperature on the acidity of CAM:MEN.

### 2.4 CO_2_ Solubility and Henry’s Law Constant

First, the experimental setup was validated by performing CO_2_ absorption studies using Reline as the absorbent (Figure S2) at 313.15 K. The results are comparable to the literature-reported CO_2_ solubility. Further, the study was continued with CAM:MEN. It is well-known that gas absorption is favored at low temperatures and high pressure. The findings in the present study support absorption thermodynamics (Figure 7). Even though the maximum solubility in CAM:MEN was observed at 298.15 K (Figure 7a), we chose 303.15 K as the optimum temperature of operation for further studies (Figure 7b) due to enhanced cooling requirements at 298.15 K. The highest (0.044 ± 0.002) and lowest (0.024 ± 0.01) CO_2_ solubilities are observed at 0.6 and 0.2 MPa, respectively. Table 3 presents the CO_2_ solubility data for several other DESs. It can be concluded that high operating pressure can significantly enhance the solubility.

**Figure 7:**
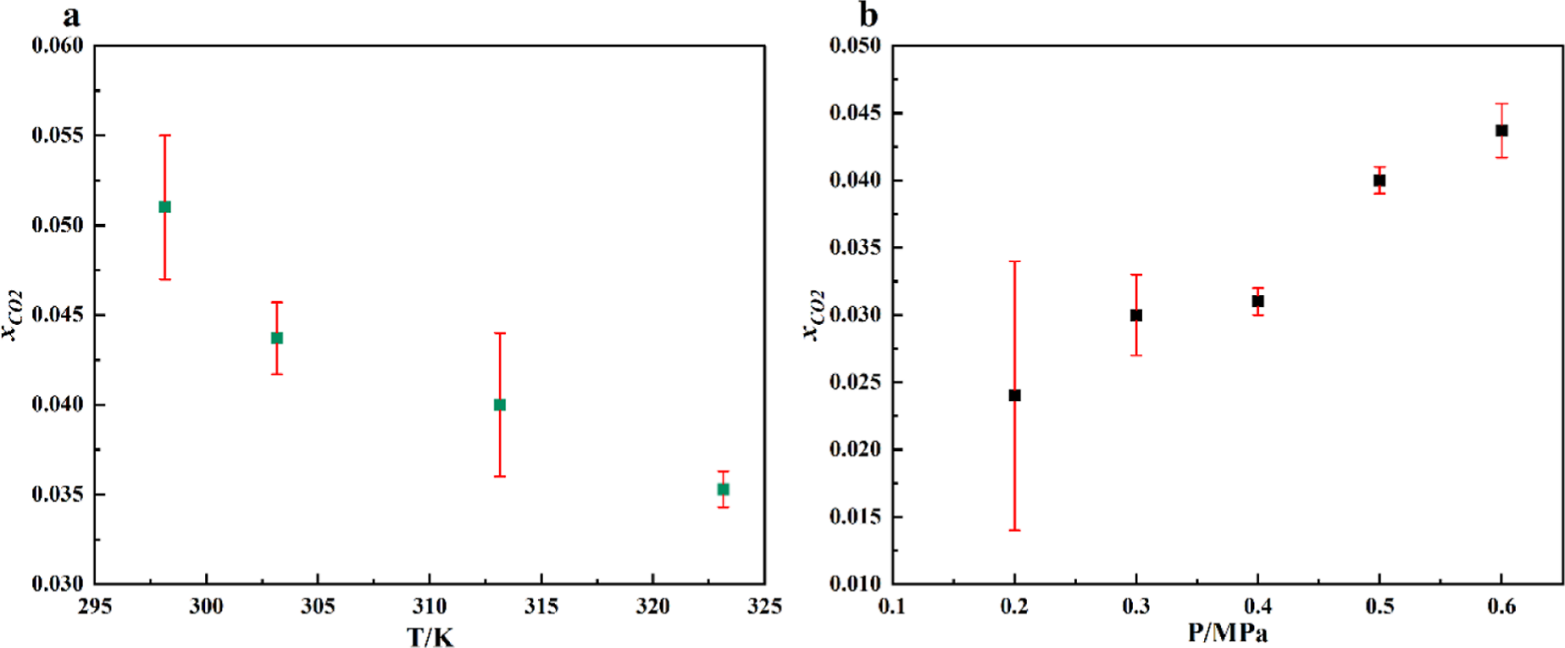
Variation in CO_2_ solubility under (a) a varying temperature range from 298.15 to 323.15 K and 0.6 MPa and (b) a pressure range of 0.2 to 0.6 MPa at 303.15 K. The red line represents the standard deviation.

**Table 3:** CO_2_ Solubility (Mole Fraction) Comparison with Literature

solvent	*x*_CO_2__	operating conditions
CAM:MEN (4:7)^[this Study]^	0.044 ± 0.002	0.6 MPa, 303.15 K
CAR:THY (1:1)^3^	0.5	4 MPa, 298.15 K
CAR:THY (1:1)^3^	0.48	3.5 MPa 298.15 K
CIN:MEN (1:1)^3^	0.51	
CIN:THY (1:1)^3^	0.5	
DecA:N_8888_Cl (1.5:1)^15^	∼0.3	2 MPa, 298.15 K
ChCl:U (1:2)^13^	0.303	12.4 MPA, 313.15 K
ChCl:U (1:2)^1^	∼0.0035	0.15 MPa, 309.15 K
ChCl:EG (1:2)^1^	∼0.0034	0.143 MPa, 309.15 K
ChCl:EG:malonic acid (1.3:1:2.2)^1^	∼0.0034	0.157 MPa, 309.15 K

As depicted in Figure 8, Henry’s law constant increases with the rise of temperature and is inversely proportional to CO_2_ solubility. This indicates an increase in the fugacity (fugacity coefficient) at elevated temperatures. The mass transfer from one phase to another is governed by the difference in fugacity. CO_2_ molecules continue to move toward the liquid phase (lower fugacity) from the gas phase (higher fugacity). Hence, the greater the difference in fugacity of the two phases, the better the absorption. As the fugacity of the solvent starts increasing, the gas solubility retards. Table S3 compares Henry’s law constant of this study with previously reported values. However, as Henry’s law constant is dependent on temperature and pressure, comparing different solvents demands identical operating conditions.

**Figure 8:**
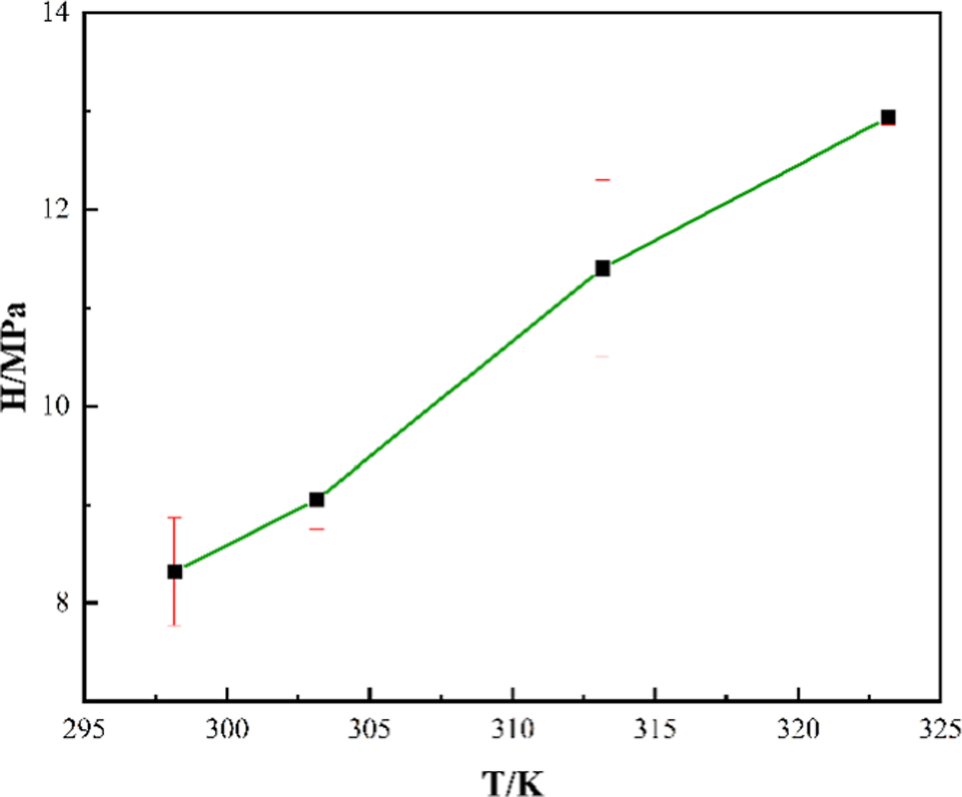
Henry’s law constant at different temperatures and 0.6 MPa.

### 2.5 Enthalpy and Entropy Change of Absorption

The changes in enthalpy (*Δ_abs_* *H*) and entropy of absorption (*Δ_abs_* *S*) were computed by using eqs 1 and 2. The variation in Henry’s law constant with temperature at 0.6 MPa data was used to calculate the properties (Table 4). The calculated enthalpy value suggests that the CO_2_ absorption is an exothermic process,^35^ which supports a reduction in CO_2_ solubility with the temperature rise. Besides that, the negative entropy value infers an ordered system. Table 5 compares the enthalpies and entropies of CO_2_ absorption in different DESs.

$$\Delta_{\mathrm{abs}}H=R\cdot {\left(\frac{\partial \ln\;H}{\partial \left(\frac{1}{T}\right)}\right)}_{P}$$ 1

$$\Delta_{\mathrm{abs}}S=\frac{\Delta_{\mathrm{abs}}H}{T}=-R\cdot {\left(\frac{\partial \ln\;H}{\partial \ln T}\right)}_{P}$$ 2

**Table 4:** Enthalpy and Entropy of CO_2_ Absorption in Different DESs

solvent system	*T*/K	*Δ_abs_H*/kJ.mol^–1^	*Δ_abs_S*/J.mol^–1^.K^–1^
CAM:MEN (4:7)^[this work]^	298.15 to 323.15	–14.7 ± 1.51	–0.15 ± 0.02
DecA:N_4444_–Cl (2:1)^15^		–11.6 ± 0.3	–37.3 ± 0.9
DecA:N_8888_–Br (2:1)^15^		–10.5 ± 0.5	–33.9 ± 1.5
DecA:N_8888_–Cl (1.5:1)^15^		–10.4 ± 0.2	–33.6 ± 0.7
DecA:N_8881_–Cl (2:1)^15^		–10.5 ± 0.3	–33.9 ± 0.9
Reline^1^	319.15	–12.59	–91.04
Ethaline^1^		11.09	–86.07
Malinine^1^		–12.65	–91.70

**Table 5:** Environmental Properties of the HBA and HBD Computed from COSMO-RS

	*S*_aq_/ mg.l^–1^		*logK*_oc_		half-time/days	
compound	298.15 K	303.15 K	298.15 K	303.15 K	298.15 K	303.15 K
MEN	47.65	54.26	2.36	2.36	1.2	1.2
CAM	1122.44	1188.8	1.66	1.66	2.5	2.5

### 2.6 ATR-FTIR, Raman Spectroscopy, and NMR Analysis

ATR-FTIR was used to compare the structures of the prepared DES at two different temperatures, i.e., 298.15 and 343.15 K, and can be found in Figure S3. It indicates presence of no new compound in the DES irrespective of the temperature of synthesis. FTIR was also performed to study the molecular structure of the HNDES formation (Figure 9a) and qualitative analysis of CO_2_ capture in HNDES. The FTIR spectrum of the before and after CO_2_ capture is reported in Figure 9b. The O–H stretching of the hydroxyl group in MEN can be observed at 3248 cm^–1^, and the C=O stretching of the carbonyl group of CAM is exhibited at 1738 cm^–1^. Spectra observed at 1100 to 1464, 2868 to 2958, 1024 to 1044, and 3248 cm^–1^ for MEN can be attributed to isopropyl, methyl, C–O stretching vibration, and hydroxyl groups, respectively.^36^

**Figure 9:**
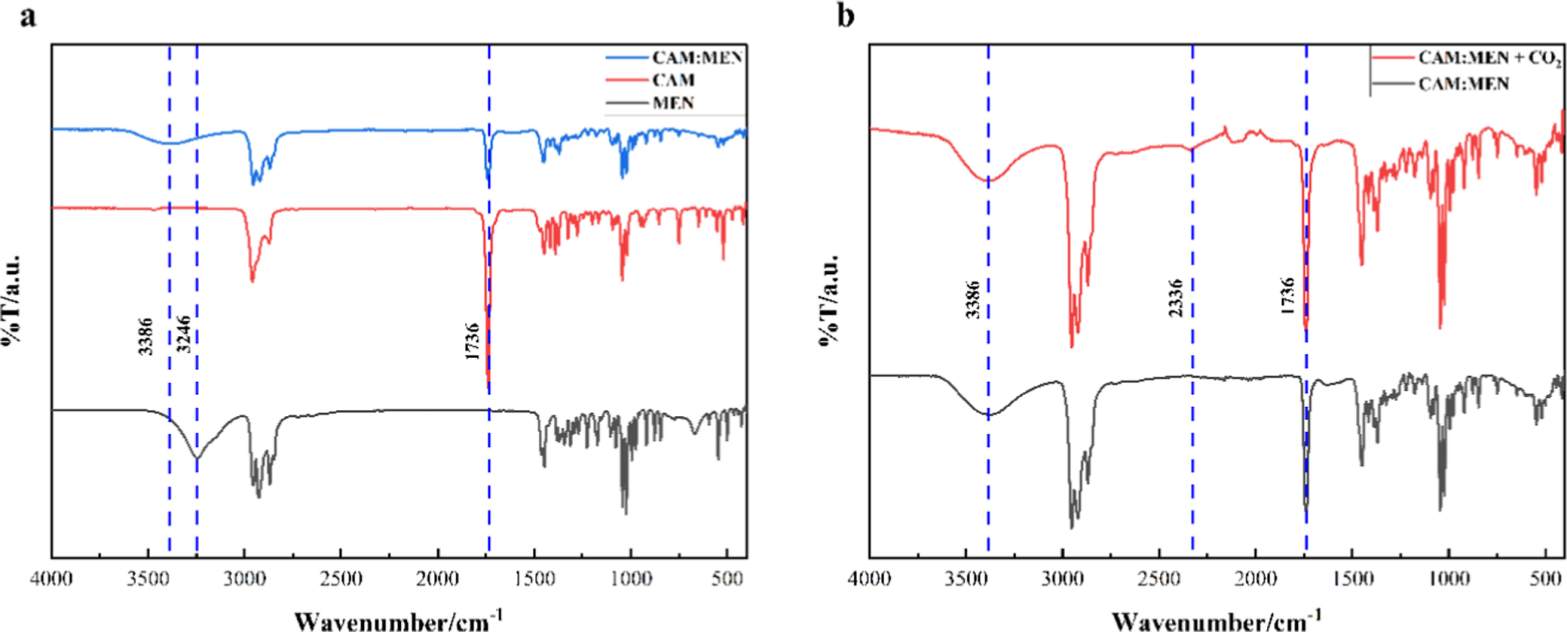
ATR-FTIR spectrum of (a) CAM:MEN and (b) before and after CO_2_ capture in CAM:MEN.

The peak for the O–H stretching of the hydroxyl group (3248 cm^–1^) in MEN is shifted to 3386 cm^–1^ after DES formation and confirms intermolecular hydrogen formation (Figure 9a). The peaks at 2868 to 2958 cm^–1^ were superimposed upon –OH stretching during the hydrogen bond formation.^36^ This can be attributed to NADES formation. Moreover, from Figure 9b, it can be observed that a peak emerges at 2336 cm^–1^ after CO_2_ absorption in HNDES, which corresponds to the asymmetric stretching of CO_2_. Similar observations were made elsewhere.^2,35^ Furthermore, there is no shifting or appearance of new peaks in the DES after the CO_2_ absorption, which indicates the absence of any chemical interaction. Literature suggests that in chemisorption, the carbamate formation peak generally appears at 1700 cm^–1^ and 2900 cm^–1^.^37,38^

The Raman spectra of CAM:MEN can be found in Figure 10. The first two characteristic peaks at 642 and 760 cm^–1^ correspond to the CAM’s and MEN’s ring deformation, respectively.^39,40^ The peak at 1451 cm^–1^ represents the C–H bending vibration of MEN.^39^ In addition, the vibrations in the isopropyl group of the MEN appear at 1172 cm^–1^.^41^ Moreover, this appearance occurs due to the rocking modes among the methyl groups of isopropyl with C–C stretches.^41^ In addition, the C=O stretching of CAM can be observed at 1731 cm^–1^.^42^ The peaks near 3000 cm^–1^ are associated with the –OH group of MEN.^43^ Interestingly, there is no new peak after CO_2_ absorption by CAM:MEN, even though the CO_2_ symmetric bond stretching is detectable by Raman. This is possibly due to the quick desorption of the gas molecules at reduced pressure before sample analysis. A study by Bhawna *et al.* observed carbamate formation in superbase-added DESs.^37^ They observed a new peak at 1100–1500 cm^–1^ (O–C–O), and the disappearance of a peak at 2900 cm^–1^ (N–H stretching) in CO_2_-captured DES. This confirmed the formation of carbamate groups (−NHCOO^–^). However, the absence of such a peak in the current work confirms the physical absorption of the CO_2_.

**Figure 10:**
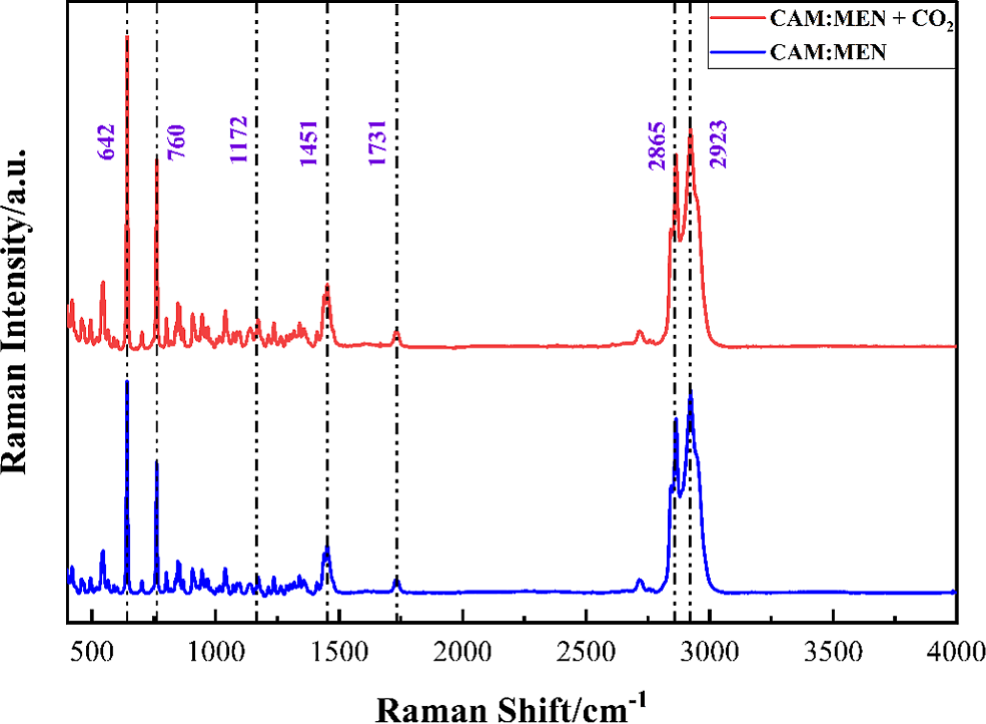
Raman spectrum of CAM:MEN before and after CO_2_ capture.

Further, ^1^H and ^13^C NMR spectra were recorded for the pure DES and DES with CO_2_ (Figures S4 and S5). Both spectra reveal the absence of carbamate formation, supporting FTIR and Raman observations regarding CO_2_ physisorption. A similar observation was made by Harifi-Mood *et al.*, in terpene-based DES for CO_2_ capture.^44^ The authors noted no significant changes in the ^1^H NMR absorption. However, in situ NMR techniques can overcome this challenge by combining ^13^C and ^17^O NMR to mark the presence of CO_2_.^45^ Moreover, carbamate formation is evident by NMR in chemisorption-based CO_2_ capture processes.^46^ Figure S6 clearly indicates that the color of the solvent remains unchanged after CO_2_ capture. Zheng *et al.* reported that milky white precipitation of carbamate occurred after absorption.

### 2.7 Regeneration Performance of HNDES

In any absorption process, the regeneration of the solvent is a crucial factor, as it determines the sustainability of the solvent on an industrial scale. The reversibility of the HNDES was examined and can be found in Figure 11. The desorption study was performed after the absorption. A total of five cycles were performed, and a loss of 5% in CO_2_ solubility was noted at the end of the fifth cycle. This indicates the high regeneration capability of the studied HNDES up to the fourth cycles.

**Figure 11:**
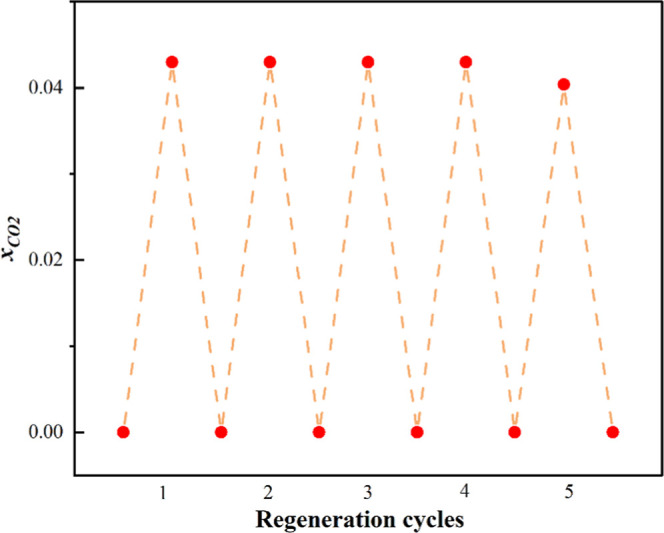
Regeneration of the CAM:MEN for five consecutive cycles (absorption at 303.15 K and desorption at 313.15 K, *P* = 0.6 MPa).

### 2.8 Environmental Screening

Besides the SLE and other property calculations, the COSMO-RS was also used for the HBA and HBD environmental screening. Aqueous solubility (*S_aq_*), soil penetration, and atmospheric lifetime are determined (Table 5).^47–50^ These properties are crucial to understand the environmental impact of the DES. The aqueous solubility of the CAM is significantly higher than that of MEN, indicating a stronger interaction with water. Further, soil penetration and half-time of both CAM and MEN are comparable. The higher the soil penetration value (*K_oc_*), the lower is the soil penetration.^51,52^ In this study, both the components possess easy mobility through soil. The properties of DES are influenced by those of HBA and HBD. Therefore, while designing a DES, components with negligible water solubility and soil penetration capacity must be chosen. The studied DES is not entirely free of environmental risks. Nonetheless, the half-time of HBA and HBD confirms a very short atmospheric lifespan. This significantly lowers the risk of pollution.

## 3 Conclusion

The present work develops a terpene-based hydrophobic natural DES for CO_2_ absorption. COSMO-RS tool was used to compute SLE calculations and other properties such as vapor pressure, boiling point, and flash point of the HNDES. It was observed that the studied DES could be prepared without elevated heating. The viscosity of the HNDES is approximately 23 mPa·s at 298.15 K. It showed a promising CO_2_ solubility up to 0.044 ± 0.002 at 303.15 K and 0.6 MPa. The calculated Henry’s law constant was 9.045 ± 0.28 MPa. Besides that, we investigated the effect of the acidity of the HNDES on the CO_2_ absorption. The low viscosity of the solvent favors its industrial application by lowering the pumping cost. In addition, the physisorption of CO_2_ molecules in the DES was validated using FTIR, Raman, and NMR. The calculated entropy and enthalpy values indicated that the absorption of CO_2_ is an exothermic and more ordered operation. Moreover, the HNDES exhibits promising solvent regeneration capacity that can be used at the industrial level. It can be summarized that the solvent can be chosen for a low-pressure and low-energy intensive CO_2_ absorption process.

## 4 Methodology

### 4.1 Computational

Following our previously reported study, the present work used COSMO-RS to determine the molar ratio of the DES.^11^ BIOVIA COSMOtherm (version: 24.0.0) was used to predict solid–liquid equilibria between HBA and HBD. The software works on the principles of COSMO-RS. It uses Gibb’s free energy of fusion (*ΔG_fus_*) and melting temperature (*T_m_*) of the pure components to compute the eutectic point of the mixture. The detailed formula can be seen in Table S1. The enthalpy of fusion (*ΔH_fus_*) and *T_m_* for solid–liquid equilibria (SLE) calculations were provided to the system, and *ΔG_fus_* was further derived using these two input parameters. Moreover, the vapor pressure, flash point, and boiling point of our studied HNDES and the environmental properties of the HBA and HBD were also calculated.

First, the COSMO files of HBA, HBD, and CO_2_ were optimized and generated in TURBOMOLE (TmoleX 2024, version 24.0.0, BIOVIA) by DFT/B3-LYP/gridm3/DFT-D2 theory. Second, they were imported to BIOVIA COSMOtherm (version: 24.0.0) for further calculations with BP_TZVP_24 parameterization (Table 1).

### 4.2 Experimental Section

#### 4.2.1 Materials

Complete specifications of the compounds used in this study are given in Table 6. The chemicals were used without any purification.

**Table 6:**
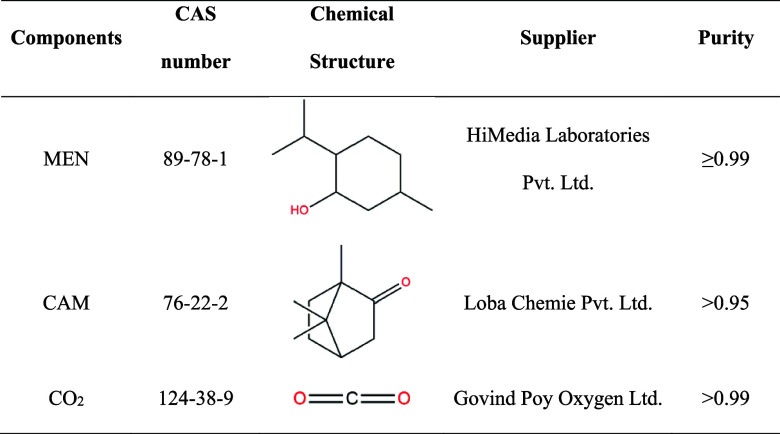
Specifications of the Chemicals Used in This Study

#### 4.2.2 Solvent Preparation

The HNDES was prepared from camphor (CAM) and menthol (MEN) at a 4:7 molar ratio following the phase diagram obtained from COSMOtherm. Both chemicals were weighed appropriately using an analytical balance (Sartorius BSEA224S-CW) with ±0.1 mg uncertainty. After that, the chemicals were transferred to a flat-bottomed flask and placed on a magnetic stirrer (REMI, 5MLH Plus). The mixture was stirred continuously at 800 rpm to obtain a clear homogeneous liquid at room temperature and atmospheric pressure.

#### 4.2.3 Solvent Characterization

HNDES formation was confirmed using analytical techniques, namely, ATR-FTIR (PerkinElmer), NMR (Bruker Avance Neo 500 MHz), and Raman (LabRam HR Horiba). Further, these techniques were also employed post-CO_2_ absorption. The FTIR spectrum was recorded from 400 cm^–1^ to 4000 cm^–1^, performing 64 scans at a resolution of 4 cm^–1^. ^1^H and ^13^C NMR studies were performed using deuterated-DMSO as the solvent. Raman spectra were captured using a He–Ne laser with an excitation wavelength of 633 nm in the range 400–4000 cm^–1^.

TGA (TA Instruments Discovery SDT 650) was performed to study the thermal behavior of the solvent. An aliquot of known weight was placed on the aluminum pan and heated from 303.15 to 573.15 K at 5 K min^–1^ under 20 mL min^–1^ N_2_ environment. The viscosity of the HNDES was measured using an Anton Paar MCR-302 rheometer with a cone plate (D-CP40-1) at a temperature range of 293–333 K and a shear stress of 1000 s^–1^. Solvent’s acidity was tested using an LMMP30 multiparameter (Labman Scientific Instruments Pvt. Ltd.) for a temperature range of 298.15 to 348.15 K. During the measurements, the temperature was maintained by a temperature-controlled water bath (DWB4, Labtical).

#### 4.2.4 CO_2_ Absorption Studies

CO_2_ absorption study was performed using a pressure drop method in a 100 mL high-pressure reactor (HPR). The HPR was manufactured and supplied by Amar Equipment Pvt. Ltd. (Model no. A1313). A PID controller was employed to control the reactor temperature, and the same was measured by a thermowell RTD PT100 sensor. Automatic internal cooling was also continuously provided by a connected serpentine cooling coil. The gas–liquid mixing was done with a 1 hp ex-proof gas group motor operated at 1000 rpm. A gas induction impeller was further used to improve the gas–liquid mass transfer rate. The gas needle valve was connected to the CO_2_ cylinder via a two-stage CO_2_ regulator. A schematic representation of the experimental setup can be found in Figure 12.

**Figure 12:**
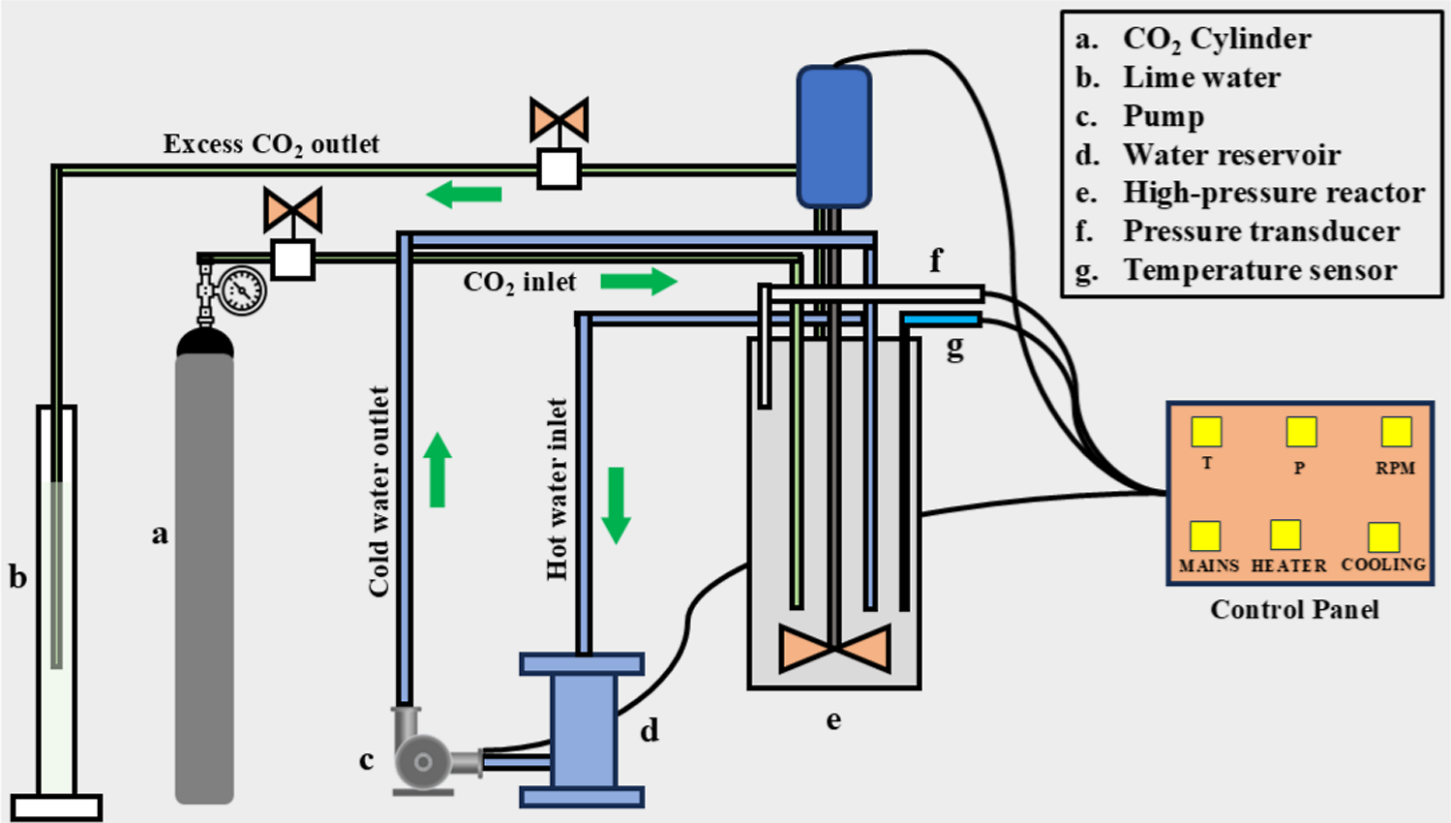
Schematic representation of the CO_2_ absorption setup.

The CO_2_ capture experiments were first performed at different temperatures (298.15–323.15 K) with a starting pressure of 0.6 MPa. This was done to identify the optimum temperature for the absorption. Nearly 25 mL of HNDES was used inside the reactor for each batch of experiments. Then, continuous stirring and heating were done until the desired temperature was achieved. Afterward, CO_2_ was purged into the reactor vessel at 0.6 MPa. The stirring process was resumed immediately after the CO_2_ purging, and the temperature was kept constant throughout the absorption process. It took nearly 12 h to attain the equilibrium of the mass transfer process. The pressure drop was monitored through the control panel until it remained unchanged for 2 h. Second, a similar experiment was carried out for a range of initial pressures (0.2–0.6 MPa) at the optimized temperature obtained from the previous set of experiments. It is observed that literature reports CO_2_ absorption at a higher pressure (1–4 MPa).^2,3,28^ All the experiments were repeated twice, and computed results are obtained by taking the average. The CO_2_ solubility is calculated in terms of the mole fraction following eq 3.

3 $$x_{{\mathrm{CO}}_{2}}=\frac{{n_{\mathrm{CO}}}_{2}}{n_{{\mathrm{CO}}_{2}}+n_{\mathrm{HNDES}}}$$

where *x_CO_2__*, *n*_HNDES_, and *n_CO_2__* denote the CO_2_ mole fraction in HNDES, the number of moles of HNDES, and the number of moles of CO_2_ absorbed in HNDES, respectively. The *n_CO_2__* and *n_HNDES_* values are calculated from eqs 4 and 5, respectively.

$$n_{{\mathrm{CO}}_{2}}=\frac{V}{R\cdot T}\left(\frac{P_{i-}P_{\mathrm{HNDES}}}{z_{i}}-\frac{P_{e}-P_{\mathrm{HNDES}}}{z_{e}}\right)$$ 4

where *V* is the gas volume, *R* is the real gas constant, *T* is the temperature inside the reactor, and *z_i_* and *z_e_* are initial and equilibrium compressibility factors, respectively. Also, *P_i_, P_e_*, and *P_HNDES_* are the initial and equilibrium pressures and vapor pressure of the HNDES. It was assumed that under specified experimental conditions, the vapor pressure of the HNDES was negligible.

5 $$n_{\mathrm{HNDES}}=\sum \left(\frac{m_{i}}{{MW}_{i}}\right)\cdot x_{i}$$

The *n*_HNDES_ is calculated from the individual mass of the components (*m*_i_), mole fraction (*x*_i_), and molecular weight (*MW*_*i*_), respectively. The Henry’s law constant was determined using the CO_2_ solubility data mentioned above, following eq 6.

$$H=\frac{f\left(P_{{\mathrm{CO}}_{2}}^{e},T\right)}{x_{{\mathrm{CO}}_{2}}}≅\frac{\Phi_{{\mathrm{CO}}_{2}}\cdot P_{{\mathrm{CO}}_{2}}^{e}}{x_{{\mathrm{CO}}_{2}}}$$ 6

where *f* is the solute fugacity at equilibrium pressure and temperature, Φ_CO_2__ is the fugacity coefficient computed from the RK equation of state (RK EoS), and $P_{{CO}_{2}}^{e}$ is the equilibrium pressure. The fugacity calculation from the RK EoS is listed in Table S2.

The same setup was used to regenerate the HNDES. The desorption process was conducted at 313.15 K with stirring at 1000 rpm.

## Supplementary Material



## Notes

Mathematical model used in the various COSMO-RS calculations, RK EoS, Henry’s constant comparison, SCD of the pure components and HNDES, calibration graph, ^1^H and ^13^C NMR spectra, FTIR spectrum, and pictures of CO_2_ absorbed solvent (PDF)
