# Supplementary material for: Terpene-Derived Low-Viscosity Deep Eutectic Solvent for Energy-Efficient CO2 Absorption
Source: ACS Omega. 2025 Sep 30;10(40):47216–26. doi: 10.1021/acsomega.5c06033 (PMC12529136; doi:10.1021/acsomega.5c06033)
Supplement: Supplementary file 1 [file ao5c06033_si_001.pdf]

Terpene-derived low-viscosity deep eutectic solvent for energy efficient CO_2 absorption

Supporting Information

Sinchan Hait[1], Upasana Mahanta[*]

[1,*] Department of Chemical Engineering, Birla Institute of Technology and Science, Pilani, K.K. Birla Goa Campus, Zuarinagar, Sancoale, Goa 403726, India.

*Corresponding Author: Upasana Mahanta,

Department of Chemical Engineering,

Birla Institute of Technology and Science, Pilani, K.K. Birla Goa Campus,

Zuarinagar, Sancoale, Goa 403726, upasanam@goa.bits-pilani.ac.in

Table S1: A thorough explanation of the mathematical techniques applied in the current work in COSMO-RS

Sr no.	Property	Mathematical model	Description
1	σ-profile	$p_S(\sigma) = \sum_{i \epsilon S} [x_i p_i(\sigma)]$	• "$p_i(\sigma)$" sigma profile of any molecule<br>• "x_i" mole fraction of component "*i*" in mixture.
2	SLE	$G_i^{solid} = G_i^{solution}$<br>$\mu_i^{pure} - max\left(0, \Delta G_{fus}(T)\right) = \mu_i^{solution} + RT \ln x_i$<br>$\Delta G_{fus}(T) = \Delta H_{fus}(1 - T/T_m) - \Delta c_p^{fus}(T_{melt} - T) + \Delta c_p^{fus} \ln(T_m/T)$	• Temperature depended "ΔG_{fus}" calculated from "ΔH_{fus}" and "T_m".<br>• "$\mu_i^{solution}$" is chemical potential of the "*i*" compounds in solution.
3	Vapor pressure	$p_i^{S,vap}/1\ bar = exp - \left[\left(G_i^{S,\infty} - G_i^{Gas}\right) / RT \right]$	• Gibbs free energy of the compound "*i*" in the gas phase ($G_i^{Gas} = \mu_i^{Gas} = E_i^{Gas}$)<br>• Gibbs free energy of the compound "*i*" in the

			liquid phase ($G_i^{S,\infty} = E_i^{COSMO} + \mu_i^{S,\infty}$)
4	Density	$\rho_i = MW_i/\tilde{V}_i N_A$	• "ρ_i" liquid density of "i" component<br>• "$\tilde{V}_i$" corrected molar volume of the compound.<br>• "MW_i" molecular weight of the compound<br>• "N_A" Avogadro's constant.
5	Water solubility	$\log S_S^X = \log\left(\frac{MW_X \rho_S}{MW_S}\right) + \frac{\ln(10)}{KT}\left[-\Delta_S^X + min\left(0, \Delta G_{fus}^X\right)\right]$<br>$\Delta_S^X = \mu_S^X - \mu_X^X$	• "MW_X" molecular weight of "X"<br>• "MW_S" molecular weight of "S".<br>• "ρ_S" density of "S".<br>• "K" Boltzman constant<br>• "Δ_S^X" is the difference between chemical potential of "X" in "S" and in pure "X".

6	Soil partition coefficient	$\log K_{OC} = 0.63(\pm 2)\log K_{OW} + 0.80(\pm 6)$	• "$\log K_{OC}$" soil sorption co-efficient.<br>• "$\log K_{OW}$" octanol-water partition coefficient
7	Atmospheric lifetime	$K_{abs}^{H} = K_{O}^{H}\left(1 + \sum_{i} w_{lp}^{i} + f_{steric}^{H,i}\right)$<br>$f_{steric}^{H,i} = exp\left\{-\left(d_{lp}(H,i) - d_0\right)^2/2t^2\right\}$	• "K_{abs}^{H}" is the intrinsic reactivity of an H-atom in H-abstraction from H-bonded OH adducts at oxygen lone-pair.<br>• "K_{O}^{H}" basic reactivity of each H-atom at an sp^3-C<br>• "w_{lp}^{i}" relative probability of lonepair to construct H-bonded OH-adducts and tendency to release from adduct<br>• "$f_{steric}^{H,i}$" steric availability of hydrogen atom H from an adduct state at lonepair "*i*".

Table S2: RK EoS for fugacity calculation

$$\ln \Phi_{CO_2} = Z - 1 - \ln(Z - B \cdot P) - \frac{A^2}{B} \cdot \ln\left(1 + \frac{B \cdot P}{Z}\right) \quad 1$$

$$P = \frac{R \cdot T}{V - b} - \frac{a}{T^{0.5} \cdot V \cdot (V + b)} \quad 2$$

$$a = \frac{0.42748 \cdot R^2 \cdot T_c^{2.5}}{P_c} \quad 3$$

$$b = \frac{0.08664 \cdot R \cdot T_c}{P_c} \quad 4$$

$$A^2 = \frac{a}{R^2 \cdot T^{2.5}} \quad 5$$

$$B = \frac{b}{R \cdot T} \quad 6$$

Where a, b, A^2, and B are the RK EoS parameters, V is the molar volume, and T_c and P_c are the critical pressure and critical temperature of CO_2, respectively.

Table S3: Henry's law constant comparison of CO_2 capture in various solvent systems at different temperatures.

Solvent	*H*/MPa		
	**298.15 K**	**303.15 K**	**308.15 K**
CAM:MEN (4:7)[This study]		9.045 ± 0.28	
DecA: N_{4444}-Cl(2:1)[1]	7.55 ± 0.08		8.74 ± 0.04
DecA: N_{8888}-Cl(2:1)[1]	6.17 ± 0.20		6.96 ± 0.07
DecA: N_{8888}-Cl(1.5:1)[1]	5.90 ± 0.09		6.55 ± 0.07
DecA: N_{8881}-Cl(2:1)[1]	7.18		8.27
DecA: N_{8881}-Br (2:1)[1]	7.15		7.98
Lactic acid: N_{4444}-Cl(2:1)[2]			14.46

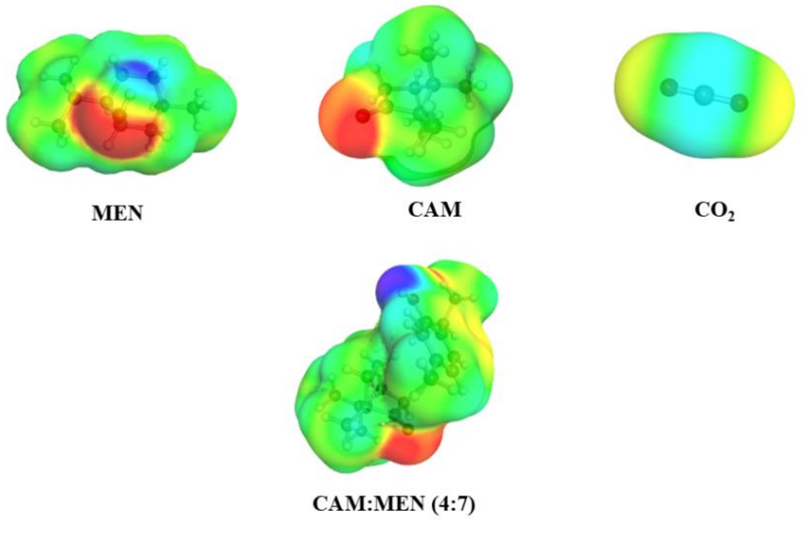

Fig. S1: SCD of MEN, CAM, CO_2, and HNDES. Red, green, yellow, and blue indicate electronegative, partially electropositive, partially electronegative, and electropositive zones, respectively.

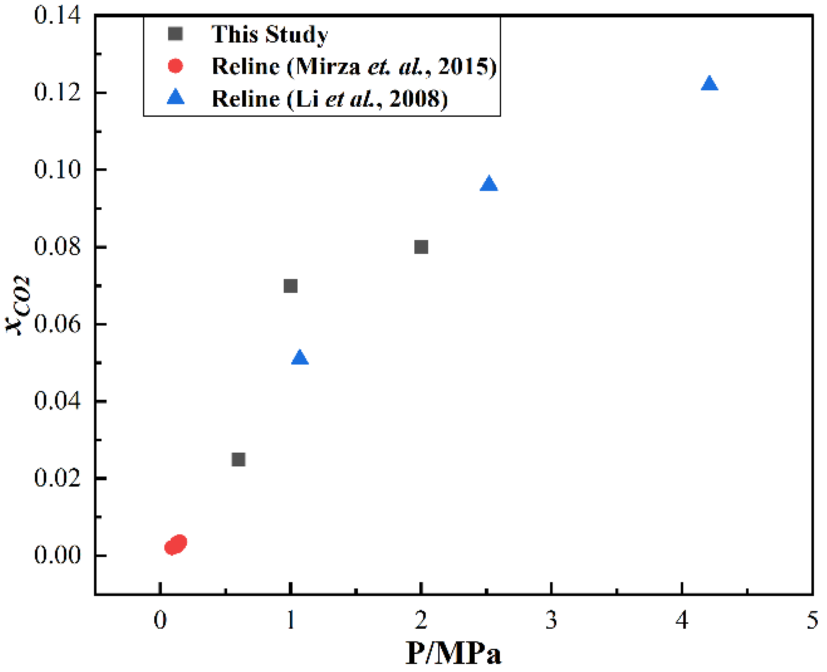

Fig. S2: Experimental setup calibration compared with the reported value in the literature.

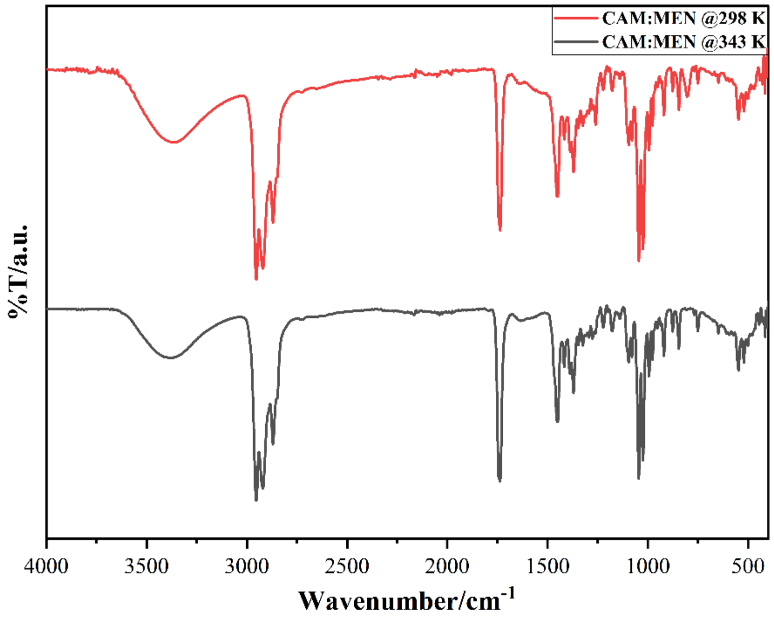

Fig. S3: FTIR spectra comparison of CAM:MEN prepared at 298.15 K and 343.15 K

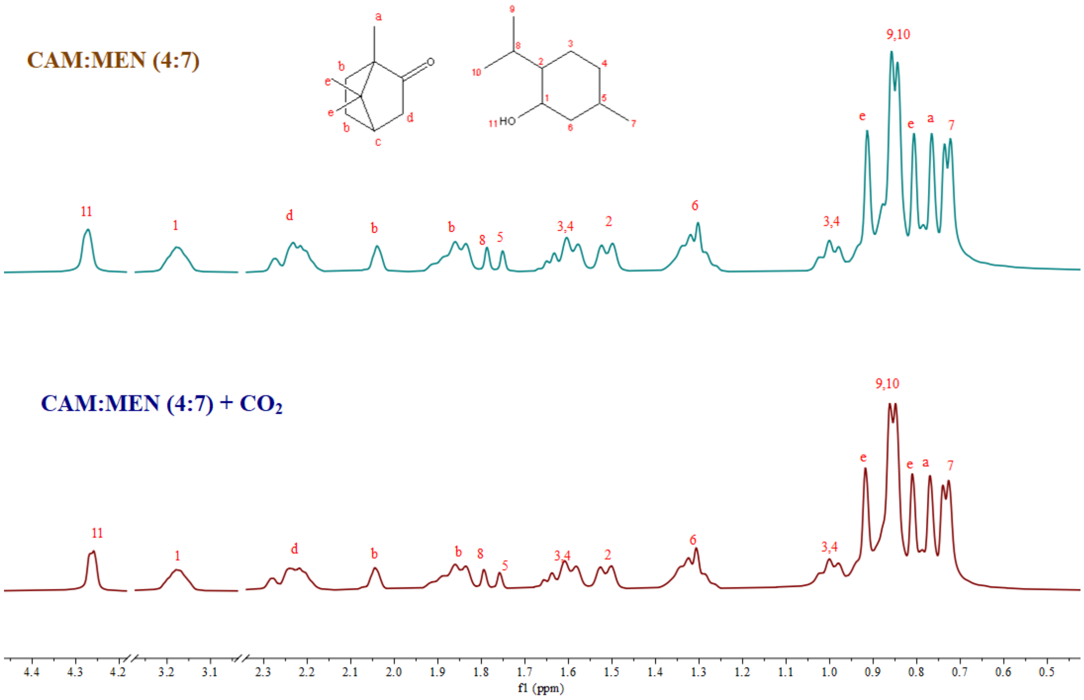

Fig. S4: ^{1}H NMR spectra of CAM:MEN (4:7), before and after CO_2 absorption.

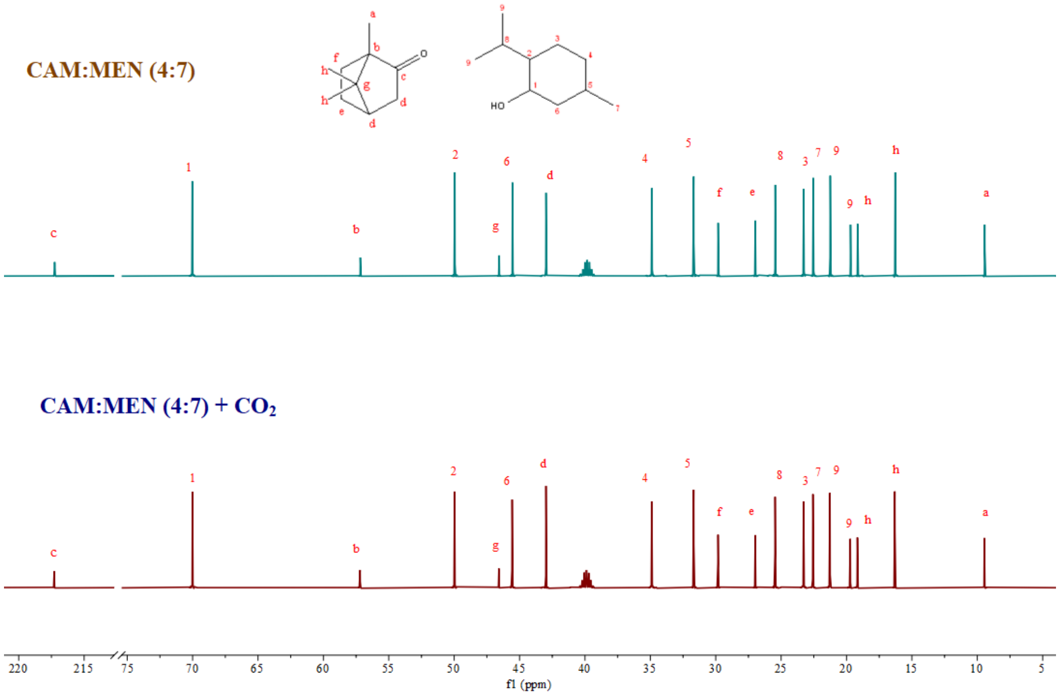

Fig. S5: ^{13}C NMR spectra of CAM:MEN (4:7), before and after CO_2 absorption.

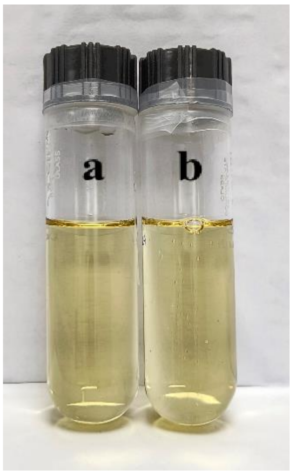

Fig. S6: CAM:MEN (4:7) before (a) and after (b) CO_2 capture.

Reference:

(1) Zubeir, L. F.; van Osch, D. J. G. P.; Rocha, M. A. A.; Banat, F.; Kroon, M. C. Carbon Dioxide Solubilities in Decanoic Acid-Based Hydrophobic Deep Eutectic Solvents. *J Chem Eng Data* **2018**, *63* (4), 913–919. https://doi.org/10.1021/acs.jced.7b00534.

(2) Zubeir, L. F.; Lacroix, M. H. M.; Kroon, M. C. Low Transition Temperature Mixtures as Innovative and Sustainable CO_2 Capture Solvents. *J Phys Chem B* **2014**, *118* (49), 14429–14441. https://doi.org/10.1021/jp5089004.

(3) Mirza, N. R.; Nicholas, N. J.; Wu, Y.; Mumford, K. A.; Kentish, S. E.; Stevens, G. W. Experiments and Thermodynamic Modeling of the Solubility of Carbon Dioxide in Three Different Deep Eutectic Solvents (DESs). *J Chem Eng Data* **2015**, *60* (11), 3246–3252. https://doi.org/10.1021/acs.jced.5b00492.
